# Long-term effects of regular exercise during pregnancy on overweight and obese gravidas: A protocol of randomized controlled trial

**DOI:** 10.1097/MD.0000000000023955

**Published:** 2021-02-05

**Authors:** Ying Zhang, Yi Yang, Guoping Xiong, Fen Yu

**Affiliations:** Department of Obstetrics, Wuhan Central Hospital, Tongji Medical College, Huazhong University of Science and Technology, Jiangan District, Wuhan City, Hubei Province, P.R. China.

**Keywords:** obese, overweight, protocol, regular exercise

## Abstract

**Background:**

Overweight before pregnancy is independent risk factor for diabetes mellitus. This randomized controlled trial was to investigate the long-term effects of regular exercise during pregnancy on overweight and obese gravidas.

**Methods and analysis:**

This single-center, prospective, randomized controlled test will be conducted in Wuhan Central Hospital, Tongji Medical College, Huazhong University of Science and Technology. Overweight and obese pregnant women will be included in our study and randomized into 2 groups: regular exercise and control groups. The informed consent will be acquired in each patient. Body weight, body fat, fasting and 2 h glucose level in 75 g oral glucose tolerance test (OGTT), insulin resistance index, and lipid profiles were compared. We also evaluated their physical activities with International Physical Activity Questionnaire (IPAQ), their dietary habits with modified Adult Dietary Behavior Assessment Scale, and depression condition with Postpartum Depression Screen Scale (PDSS). The significance level was defaulted as *P* < .05.

**Results:**

Results will be published in relevant peer-reviewed journals.

**Conclusion:**

Our study aims to systematically assess the effects of regular exercise for overweight and obese gravidas, which will be provided clinical guidance for overweight and obese gravidas.

## 1 Introduction

With the development of social economy, the incidence of overweight and obesity is increasing globally and has become the primary disease burden.^[1,2]^ According to 2013 estimates of global burden of disease (GBD), the prevalence of overweight and obesity in boys in developed countries is 23.8% and that in girls is 22.6%.^[3]^ Mean body mass index (BMI), overweight and obesity are increasing world-wide due to changes in diet and physical inactivity.^[4]^ At least 2.8 million people die each year as a result of obesity.^[5,6]^ Overweight and obesity before pregnancy are independent risk factors for gestational diabetes.^[7]^ The risk of gestational diabetes mellitus (GDM) is increased as pre-pregnancy BMI increasing, and the risk of GDM increases significantly in women with pre-pregnancy overweight or obesity.^[8]^ It also indicated that regular exercise during pregnancy can effectively reduce the risks of gestational diabetes in overweight and obese pregnant women.

In current literature, there are limited randomized studies that specifically evaluate the results of regular exercise during pregnancy on overweight and obese gravidas. We thus designed a prospective, randomized controlled trial of 2 groups of patients treated with regular exercise and placebo for overweight and obese gravidas. Our goal was to compare functional outcomes, complications, and imaging features between the 2 groups.

## 2 Methods and analysis

### 2.1 Participants

Patients with these conditions will be included: age between 25 and 35 years; are able to communicate normally and agree to participate in our study; with high body mass index before pregnancy (BMI ≥ 24.0 kg/m^2^). The excluding criteria includes pregnancy again and other reason could not continue participant into the experiment.

### 2.2 Study design

This single-center, prospective, randomized controlled test will be conducted in Wuhan Central Hospital, Tongji Medical College, Huazhong University of Science and Technology. Consecutive patients with overweight and obese gravidas will be stochastic to be dealt with a regular exercise or placebo. The protocol was approved by ethics committee of Wuhan Central Hospital, Tongji Medical College, Huazhong University of Science and Technology, with the number WHCH-T-C-2020-0522. The informed consent will be acquired in each patient. This current trial protocol was recorded in the Research Registry, with the number researchregistry6264.

### 2.3 Randomization and blind

**Figure 1:**
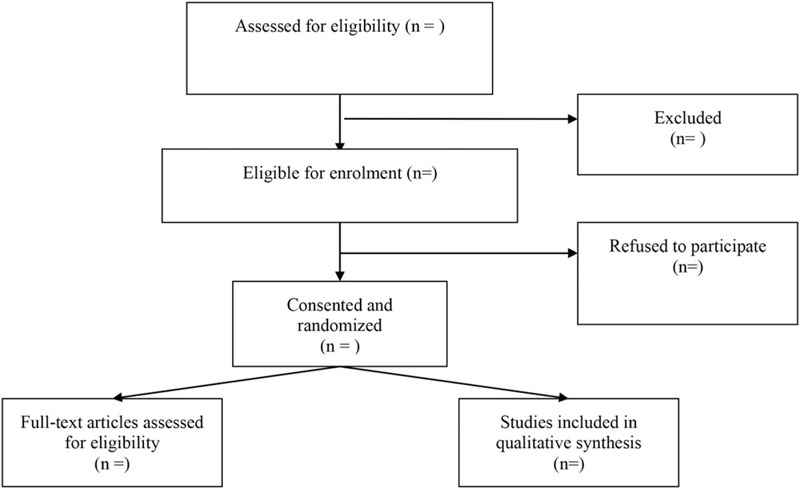
The flow diagram of procedure to select studies.

After baseline estimation and data capture, patients were stochastically divided into 2 groups by using a computer-created stochastic number table: regular exercise and placebo. Before the surgical operation, the circulating nurse will review the stochastic number table. Patients with even numbers were arranged to the regular exercise group and the patients with odd numbers were arranged to the control group. The closed envelope contains the process of treatment assignments. During the whole study, all the researchers could not see the specific grouping of treatment assignments (Fig. 1).

### 2.4 Outcome assessment

Outcomes were as follows: body weight, body fat, fasting and 2 h glucose level in 75 g oral glucose tolerance test (OGTT), insulin resistance index, and lipid profiles were compared. We also evaluated their physical activities with International Physical Activity Questionnaire (IPAQ), their dietary habits with modified Adult Dietary Behavior Assessment Scale, and depression condition with Postpartum Depression Screen Scale (PDSS).

### 2.5 Data analysis

SPSS 25.0 software for statistical analysis of data, the result of a continuous variable that is approximately normally distributed is expressed as means ± standard deviation, All data were performed using 2 independent sample *t* test; continuous variable results of non-normal distribution was expressed as median (P25–P75). The results of categorical variables are expressed by the number of cases and percentages, using the chi-squared test. *P* < .05 is considered statistically significant.

## 3 Discussion

The purpose of this randomized controlled trial was to conclude the effect of regular exercise on the long-term effect of pregnancy on overweight and obese gravidas. This study has some highlights. First, this is the first randomized controlled trial about the regular exercise in overweight and obese gravidas pregnancy. In addition, we comprehensively compare the short-term and long-term effects of regular exercised. These outcomes demonstrate the reliability of our study. Moreover, we performed rigorous statistically analysis to increase the reliability of our study. Finally, we could provide evidence for clinical guidance.

## Acknowledgment

The authors would thank for the Research Registry platform for registry for this study.

## Author contributions

**Data curation:** Ying Zhang.

**Supervision:** Ying Zhang, Yang Yi, Guoping Xiong.

**Software:** Yang Yi.

**Validation:** Guoping Xiong, Fen Yu.

**Writing – original draft:** Fen Yu.

**Writing – review & editing:** Fen Yu.

## Correction

Yi Yang's name was originally published incorrectly as Yang Yi and has since been corrected. The affiliations have also been corrected so that all authors are now affiliated with affiliation a.
